# Design, Rationale, and Methods of the INITIATE-HFrEF Trial: ARNI and SGLT2 Inhibitors Sequencing in HFrEF

**DOI:** 10.1016/j.jacadv.2026.102589

**Published:** 2026-02-04

**Authors:** João Pedro Ferreira, Ana Cristina Oliveira, Francisca Saraiva, Inês Pereira-Sousa, Rita Azevedo, António Angélico-Gonçalves, Carlos Grijó, Mariana Pereira Santos, Cristina Gavina, Mário Santos, Ricardo Fontes-Carvalho, Joana Pimenta, Adelino Leite-Moreira, Francisco Vasques-Nóvoa, INITIATE-HFrEF Investigators

**Affiliations:** a RISE-Health, Departamento de Cirurgia e Fisiologia, Faculdade de Medicina, Universidade do Porto, Porto, Portugal; b Université de Lorraine, INSERM, Centre d'Investigations Cliniques 1433, CHRU de Nancy, Inserm, 1116 and INI-CRCT (Cardiovascular and Renal Clinical Trialists) F-CRIN Network, Nancy, France; c Serviço de Medicina Interna, Unidade Local de Saúde Gaia/Espinho, Gaia, Portugal; d Escola Superior de Saúde do Porto, Instituto Politécnico do Porto, Porto, Portugal; e Serviço de Cardiologia, Unidade Local de Saúde Gaia/Espinho, Gaia, Portugal; f Serviço de Medicina Interna, Unidade Local de Saúde São João, Porto, Portugal; g Serviço de Cardiologia, Unidade de Doença Vascular Pulmonar, Unidade Local de Saúde Santo António, Porto, Portugal; h Serviço de Cardiologia, Unidade Local de Matosinhos, Matosinhos, Portugal; i Department of Immuno-Physiology and Pharmacology, Unidade Multidisciplinar de Investigação Biomédica (UMIB), Instituto de Ciências Biomédicas Abel Salazar (ICBAS), Universidade do Porto, Porto, Portugal; j Laboratory for Integrative and Translational Research in Population Health (ITR), Porto, Portugal; k Serviço de Cirurgia Cardio-Torácica, Unidade Local de Saúde São João, Porto, Portugal

**Keywords:** angiotensin-receptor neprilysin inhibitor, heart failure with reduced ejection fraction, sodium glucose co-transporter 2 inhibitor, treatment sequencing

## Abstract

**Background:**

Angiotensin receptor-neprilysin inhibitors (ARNi) and sodium glucose co-transporter 2 inhibitors (SGLT2i) are backbone guideline-directed medical therapies for patients with heart failure with reduced ejection fraction (HFrEF). Expert documents suggest that ARNi and SGLT2i should both be started rapidly. However, to date, no studies have assessed the efficacy and safety of these therapies when started simultaneously or sequentially.

**Objectives:**

The purpose of this study was to evaluate the efficacy and safety of simultaneous vs sequential initiation of ARNi and SGLT2i in HFrEF.

**Methods:**

Randomized noninferiority trial comparing 2 treatment strategies: 1) simultaneous ARNi/SGLT2i initiation (up to 5 days apart); vs 2) sequential initiation of one agent followed by the other (4-12 weeks later). The primary endpoint is a composite of hypotension, hyperkalemia, hypokalemia, estimated glomerular filtration rate drop ≥50% from baseline, emergency visit for HF, HF hospitalization, or cardiovascular death. The prespecified noninferiority margin for the absolute between-group difference is 20% with a two-sided 95% CI. Follow-up time was approximately 24 weeks.

**Results:**

Sixty-two patients were randomized: 29 simultaneous vs 33 sequential. Baseline characteristics were overall well balanced between groups. The mean age was 68.1 ± 10.4 years, 65% were men, 48% were inpatients, 79% had a “de novo” HF diagnosis, 39% of patients had a prior treatment with angiotensin-converting enzyme inhibitors/angiotensin receptor blockers, 61% with beta-blockers, and 27% with mineralocorticoid receptor antagonists. The mean left ventricular ejection fraction was 33% ± 9%, systolic blood pressure was 125.3 ± 17.4 mm Hg, estimated glomerular filtration rate was 78.1 ± 23.8 mL/min/1.73 m^2^, and serum potassium was 4.3 ± 0.5 mmol/L. Most patients in the sequential group (n = 27) were started on SGLT2i and 6 were started on ARNi.

**Conclusions:**

Initiation of ARNi and SGLT2i in Patients With HFrEF is a randomized trial to test whether a simultaneous initiation of ARNi and SGLT2i is noninferior to a sequential initiation of one agent followed by the other 4 to 12 weeks apart.

Angiotensin receptor-neprilysin inhibitors (ARNi) and sodium glucose co-transporter 2 inhibitors (SGLT2i) are backbone guideline-directed medical therapies (GDMTs) for patients with heart failure with reduced ejection fraction (HFrEF).^1^, ^2^, ^3^, ^4^, ^5^ Although the optimal timing for initiation of these therapies is undefined, expert opinion documents suggest that ARNi and SGLT2i should both be started rapidly (“within 2-4 weeks”).^6^^,^^7^ This rapid sequencing of GDMT contrasts with the historical practice of adding agents sequentially in the chronological order of pivotal clinical trials. However, in routine care, many patients already receive HF therapies even if for indications other than HF (eg, hypertension, atrial fibrillation, coronary artery disease, diabetes); therefore, “real-world” HF therapy sequencing is more nuanced and must be adapted to the clinical context of each patient and health care system.^8^ More recently, SGLT2i and ARNi both showed to improve HF outcomes among patients admitted to the hospital for worsening HF.^9^^,^^10^ A simultaneous initiation of ARNi and SGLT2i may accelerate symptom improvement, deliver earlier clinical benefit, and counteract therapeutic inertia. Still, clinicians may be reluctant to start both therapies simultaneously due to concerns about adverse events (eg, hypotension, hyperkalemia, and worsening kidney function [WKF]). Whether is safe or not to start ARNi and SGLT2i simultaneously remains undefined.

The STRONG-HF (Safety, Tolerability and Efficacy of Rapid Optimization, Helped by NT-proBNP testing of Heart Failure Therapies; NCT03412201) trial showed that a high-intensity strategy of rapid up-titration of GDMT and a close follow-up, with 4 outpatient visits within 2 months after discharge, was superior to standard of care, despite more frequent adverse events.^11^ However, STRONG-HF’s high-intensity care strategy may not be replicable in most health care systems, did not include SGLT2i as part of treatment strategy, and ARNi were used only in a small proportion of patients (<10%). Moreover, available subgroup analyses of SGLT2i use on top of ARNi were restricted to patients already receiving ARNi chronically, precluding evaluation of simultaneous initiation.^12^^,^^13^

To date, no randomized studies have compared the efficacy and safety of a simultaneous vs (vs a sequential ARNi and SGLT2i initiation. To address this knowledge gap, we designed and conducted the INITIATE-HFrEF (Initiation of ARNi and SGLT2i in Patients With HFrEF; NCT05989503) trial to compare 2 treatment strategies: 1) simultaneous initiation of ARNi and SGLT2i; vs 2) sequential initiation, in which one agent (either an ARNi or SGLT2i) is started first and the other is added within 4-12 weeks. Herein, we present the Methods and Baseline characteristics of the INITIATE-HFrEF trial.

## Methods

INITIATE-HFrEF is a low-interventional, pragmatic prospective randomized open-label investigator-initiated and investigator-led trial comparing 2 treatment strategies: 1) a simultaneous strategy with concomitant ARNi and SGLT2i initiation (up to a maximum of 5 days apart), and 2) a sequential strategy with either ARNi or SGLT2i initiation first followed by the other agent within 4 to 12 weeks.

The trial is registered on ClinicalTrials.gov (NCT05989503) and EU-CT (European Union Clinical Trials) (No. 2022-502409-14-00) and was approved by the CEIC (Portuguese Ethics Committee for Clinical Research) and the INFARMED (National Authority of Medicines and Health Products). The study was conducted in accordance with Good Clinical Practice and the Declaration of Helsinki. The trial was funded by Novartis and sponsored by the Faculty of Medicine of the University of Porto. An independent Data and Safety Monitoring Committee (CRU-RISE [Clinical Research Unit RISE]) oversaw the trial.

The first draft of the protocol was written in 2021, before the publication of EMPULSE (A Study to Test the Effect of Empagliflozin in Patients Who Are in Hospital for Acute Heart Failure; NCT04157751).^10^ At that time, most physicians in Portugal were initiating ARNi for HFrEF during hospitalization or at discharge based on the results from PIONEER-HF (Comparison of Sacubitril/Valsartan vs Enalapril on Effect on NT-proBNP in Patients Stabilized From an Acute Heart Failure Episode; NCT02554890).^9^ Consequently, we initially conceived the sequential strategy as ARNi initiation and up-titration over 12 weeks, followed by SGLT2i initiation. This strategy was specified in the first protocol version, signed in January 2023. That version circulated internally and underwent minor amendments mainly related to safety information and inclusion/exclusion criteria. The second protocol version of the protocol (March 2023) was the one submitted to regulatory authorities.

However, this “ARNi-first, SGLT2i-later” sequential strategy quickly proved impractical, and recruitment was slow. After the publication of EMPULSE in 2022, investigators were routinely initiating SGLT2i early in most patients.^10^ In parallel, uptake of SGLT2i in Portugal was rapid and widespread, at least in tertiary centers, so that many patients with HF were already receiving SGLT2i at baseline, and even patients without HF were frequently treated with SGLT2i for diabetes or chronic kidney disease.^14^ In response, we submitted a first substantial modification in the second half of 2023 with 2 key changes: 1) in the sequential arm, patients could initiate an SGLT2i first, with ARNi added between 4 (visit 2) and 12 (visit 3) weeks later; 2) in the simultaneous arm, ARNi and SGLT2i could be prescribed within a 5-day window.

A second substantial modification was submitted in the first half of 2025. The main change was a reduction in planned sample size from 172 to 62, achieved by widening the noninferiority margin from 15% to 20% and lowering the power (beta) from 90% to 80% (see the Statistical considerations section). The 20% noninferiority margin reflected a compromise between a feasible sample size and a clinically interpretable margin, as we considered that a one-sided 97.5% noninferiority margin >20% would undermine the clinical interpretability of the study. The final protocol version (V4) was approved in May 2025.

Patient enrollment started in August 2023. Recruitment ended in December 2024, and the last patient last visit was held in July 2025.

### Entry criteria and randomization

INITIATE-HFrEF enrolled adult (≥18 years) inpatients or outpatients with symptomatic HF (NYHA functional class II to IV), a left ventricular ejection fraction (LVEF) < 50% (within 12 weeks of screening for outpatients and within 2 weeks of screening for inpatients), an estimated glomerular filtration rate (eGFR) ≥25 mL/min/1.73 m^2^ (CKD-EPI [Chronic Kidney Disease Epidemiology Collaboration] 2021 creatinine-based formula), serum potassium ≤5.4 mmol/L, and systolic blood pressure (SBP) ≥100 mm Hg. Patients could not have been treated with an ARNi or SGLT2i within the previous 30 days. Prior use of angiotensin-converting enzyme inhibitors or angiotensin receptor blockers was allowed and could be maintained until switching to ARNi. Patients with a myocardial infarction, stroke, sepsis, or surgical procedure within the previous 30 days or reported intolerance to ARNi or SGLT2i were excluded.

A randomization list was generated with treatments assigned at random using a sealed envelope without stratification factors. Envelopes were prepared by the study manager in the beginning of the trial and were sealed. Physicians did not participate in this task and were unaware of future assignments. Envelopes were prepared on the day the patient was randomized by the research team.

### ARNi and SGLT2i dosage

Both empagliflozin and dapagliflozin were allowed as SGLT2i at a dose of 10 mg/day, including in combined therapies with metformin as long as the daily dose was 10 mg/day. Sacubitril/valsartan was used as ARNi with dose ranges from 24/26 mg/twice daily to 97/103 mg/twice daily. The proposed initial dose was 24/26 mg twice daily or 49/51 mg twice daily and titrated to 97/103 mg twice daily (if tolerated), preferentially within 3 to 6 weeks; still, ARNi dose adjustments were ultimately left at the discretion of the investigators.

### Study visits and procedures

Follow-up included visits at randomization (visit 1; V1), week 4 (±15 days) (V2), week 12 (±15 days) (V3), and week 24 (±15 days) (V4). Evaluations performed at each study visit are described in the protocol available in the Supplementary Appendix. Planned standard of care appointments were maintained and were performed according to physician decision. After the completion of the trial, all HF treatments were left at the discretion of the assistant physicians. Blood pressure and laboratorial measurements of potassium and creatinine were performed serially at study visits to capture prespecified safety events including hypotension, hyperkalemia, and WKF.

Patients were enrolled at four centers (*Unidade Local de Saúde*, ULS) in the North of Portugal: 1) *ULS São João, Porto*; 2) *ULS Gaia/Espinho, Vila Nova de Gaia*; 3) *ULS Santo António, Porto*; and 4) *ULS Matosinhos, Matosinhos*.

### Study objectives, comparator arm, and outcomes

The primary objective is to compare the efficacy and safety of a simultaneous vs sequential ARNi and SGLT2i treatment strategy for HFrEF over approximately 6 months of follow-up. We hypothesize that a simultaneous initiation of ARNi and SGLT2i is noninferior to a sequential initiation of these agents. We chose the sequential initiation of these agents as the “standard” comparator arm, as this approach was the most commonly used in our clinics, that is, before INITIATE-HFrEF was designed, patients did not start an SGLT2i and an ARNi simultaneously.

We thought that a noninferiority framework would be the most appropriate for this study because our outcome was mostly focused on “safety” measures (eg, hypotension, hyperkalemia, WKF) that would constitute most of the primary outcome components and, therefore, drive the treatment effect estimates on the primary outcome. If a simultaneous initiation of an ARNI with an SGLT2i would be noninferior to the more commonly used sequential approach, then this would support the simultaneous initiation of both agents as being similarly safe to the sequential approach.

The primary outcome is a composite of hypotension (SBP <100 mm Hg with signs or symptoms), hyperkalemia (serum potassium >6.0 mmol/L), hypokalemia (serum potassium <3.0 mmol/L), WKF (eGFR ≥50% drop from baseline or eGFR <15 mL/min/1.73 m2 or renal transplant or dialysis), outpatients worsening HF, emergency room (ER) visit due to worsening HF with use of intravenous (IV) diuretics, HF hospitalization or death from cardiovascular causes, analyzed in a time-to-first event manner.

The modified primary outcome is a composite of hypotension (SBP <100 mm Hg or investigator-reported hypotension), hyperkalemia (serum potassium >6.0 mmol/L or investigator-reported hyperkalemia), hypokalemia (serum potassium <3.0 mmol/L or investigator-reported hypokalemia), WKF (eGFR ≥50% drop from baseline or eGFR <15 mL/min/1.73 m^2^ or renal transplant or dialysis or investigator-reported WKF), ER visit due to worsening HF with use of IV diuretics, HF hospitalization or death from cardiovascular causes, analyzed in a time-to-first event manner.

In a nonconformity with the ClinicalTrials.gov NCT05989503 registry, we included investigator-reported outcomes as part of our modified primary outcome. This was because many patients were bringing lab results from their primary care physicians and/or had reports from other hospitals and/or had unplanned visits to the clinics without standardized clinical registries; therefore, we decided that it was more reflective of clinical practice to include all events ascertained as clinically relevant by the investigators instead of only those confirmed by our central lab or at standardized study visits or hospitalizations.

A nonprespecified main secondary outcome is a composite of hypotension (SBP <100 mm Hg or investigator-reported hypotension), hyperkalemia (serum potassium >5.5 mmol/L or investigator-reported hyperkalemia), WKF (eGFR ≥40% drop from baseline or eGFR <15 mL/min/1.73 m2 or renal transplant or dialysis or investigator-reported WKF), ER visit due to worsening HF with use of IV diuretics, HF hospitalization or death from cardiovascular causes, analyzed in a time-to-first event manner. This endpoint was created to capture more subtle, but still clinically important, potassium increments and eGFR decline compared to the primary endpoint.

Additionally, other secondary outcomes include the individual components of the primary and main secondary outcomes, diastolic blood pressure <60 mm Hg, and continuous changes overtime in SBP, eGFR, serum potassium, LVEF, body weight, uric acid, hemoglobin, natriuretic peptides (NPs), and health status assessed using the Kansas City Cardiomyopathy Questionnaire-12 item. An echocardiogram was performed at baseline (if no prior echocardiogram was available or if the screening echocardiogram had been performed more than 24 weeks for outpatients or 2 weeks for inpatients prior to randomization) and at the end of study (V4). As N-terminal-pro brain natriuretic peptide (NT-pro BNP) and BNP were measured in different centers, we standardized the NP values to all have a mean of zero and an SD of one. Therefore, each patient’s value on the standardized variable indicates the difference from the mean of the original variable in number of SDs (of the original variable).

Follow-up patterns, study visits, blood sample analyses, methods of blood pressure measurements, and background routine care were similar in both treatment groups.

### Statistical considerations

Assuming a primary outcome cumulative risk of 30% in the sequential group at 6 months, if a true difference between the intervention groups of 10% or greater exists (eg, 40% in simultaneous vs 30% in sequential), then 62 patients (31 per group) would be required to have 80% power for the upper limit of a two-sided 95% CI to exclude a difference in favor of the sequential group >20%. Descriptive statistics were used to describe the study population overall and by treatment allocation group. The primary composite outcome, main secondary outcome, and their individual components were analyzed by means of a Cox model with the time-to-first event as outcome and treatment allocation (simultaneous vs sequential) as independent variable, in an intention-to-treat manner. We did not perform covariate adjustment to avoid model overfitting after the sample size reduction. Absolute differences were calculated using the Kaplan-Meier method of cumulative incidence difference at the end of follow-up. Changes in continuous variables over time were studied using mixed models for repeated measures using the continuous variable of interest as outcome, treatment (simultaneous vs sequential), visit (V1, V2, V3, V4), treatment-by-visit interaction, and the baseline value of the variable of interest as independent variables, and random effects by patient *id*. No interim analysis was performed. Analyses were conducted using Stata (StataCorp. 2023. *Stata Statistical Software: Release 18*. StataCorp LLC). A *P* value <0.05 was considered statistically significant. No data imputation was performed. No multiple test correction was performed for secondary outcomes or subgroup analyses.

## Results

### Baseline patient’s characteristics

**Figure 1:**
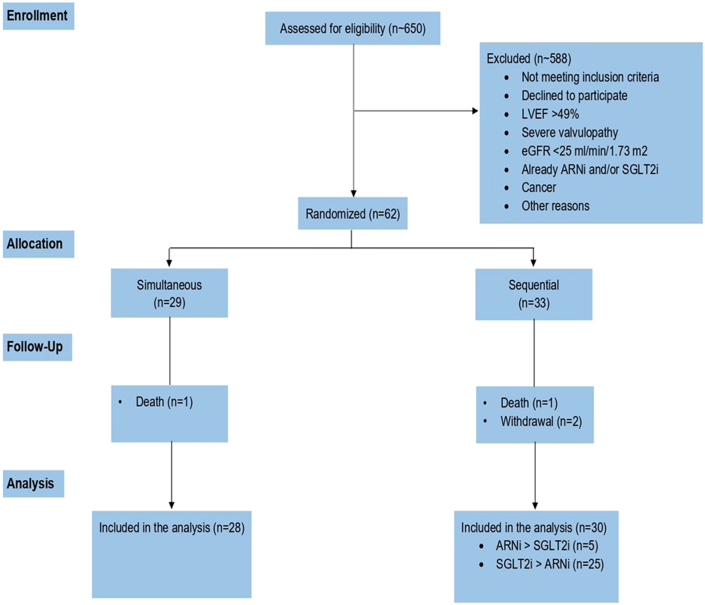
**Study Flowchart** SGLT2i = sodium glucose co-transporter 2 inhibitor; ARNi = angiotensin-receptor neprilysin inhibitor; LVEF = left ventricular ejection fraction; eGFR = estimated glomerular filtration rate.

A total of 62 patients were randomized: 29 simultaneous vs 33 sequential. The study flowchart is presented in Figure 1.

Fifty-eight patients completed the study, 2 patients had deceased (1 per group, noncardiovascular causes, only performed 1 and 2 visits), and 2 sequential patients withdrawn (patient initiative). The mean age was 68.1 ± 10.4 years, 65% were men, 48% were inpatients, 79% had a “de novo” HF diagnosis, 31% had atrial fibrillation or flutter on electrocardiogram, 26% had diabetes, 39% of patients had a prior treatment with angiotensin-converting enzyme inhibitors or angiotensin receptor blockers, 61% with beta-blockers, and 27% with mineralocorticoid receptor antagonists. The mean LVEF was 33 ± 9%, SBP was 125.3 ± 17.4 mm Hg, eGFR was 78.1 ± 23.8 mL/min/1.73 m^2^, and serum potassium was 4.3 ± 0.5 mmol/L. The randomized treatment groups were overall well-balanced, with exception of body mass index and NPs which were higher and lower in the sequential group, respectively (*P* < 0.05 for both). Most patients in the sequential group (n = 27) were started on SGLT2i and 6 were started on ARNi (Table 1).

**Table 1:** Baseline Patient Characteristics

	Overall (N = 62)	Simultaneous (n = 29)	Sequential (n = 33)	Missing
Age, y	68.1 ± 10.4	67.5 ± 10.0	68.6 ± 10.8	0
Men	40 (65%)	19 (66%)	21 (64%)	0
SGLT2i	56 (90%)	29 (100%)	27 (82%)	-
None	6 (10%)	0 (0%)	6 (18%)	
Dapagliflozin	39 (63%)	21 (72%)	18 (55%)	
Empagliflozin	17 (27%)	8 (28%)	9 (27%)	
ARNi	35 (56%)	29 (100%)	6 (18%)	-
24/26 mg twice daily	23 (66%)	21 (72%)	2 (33%)	
49/51 mg twice daily	12 (34%)	8 (28%)	4 (67%)	
Inpatient	30 (48%)	13 (45%)	17 (52%)	0
New-onset HF	49 (79%)	24 (83%)	25 (76%)	0
Ischemic HF	17 (27%)	8 (28%)	9 (27%)	0
Prior HF hosp.	5 (8%)	2 (7%)	3 (9%)	0
AFib/flutter (ECG)	19 (31%)	8 (28%)	11 (33%)	0
Diabetes	16 (26%)	6 (21%)	10 (30%)	0
Smoking	18 (29%)	8 (28%)	10 (30%)	0
NYHA functional class				1 (1.6%)
II	48 (79%)	24 (83%)	24 (75%)	
III/IV	13 (21%)	5 (17%)	8 (25%)	
Pulmonary rales	29 (48%)	15 (52%)	14 (44%)	1 (1.6%)
Peripheral edema	12 (20%)	4 (14%)	8 (25%)	1 (1.6%)
BMI, kg/m^2^^a^	26.4 ± 6.9	24.2 ± 6.9	28.4 ± 6.3	0
SBP, mm Hg	125.3 ± 17.4	124.8 ± 15.0	125.7 ± 19.4	0
DBP, mm Hg	73.7 ± 13.8	74.2 ± 13.6	73.2 ± 14.2	0
Heart rate (ECG), beats/min	75.2 ± 16.7	78.2 ± 16.6	72.4 ± 16.5	1 (1.6%)
LVEF, %	32.8 ± 8.9	33.0 ± 8.8	32.6 ± 9.0	0
Hemoglobin, g/dL	13.5 ± 2.0	13.4 ± 2.1	13.6 ± 1.9	0
Creatinine, mg/dL	0.9 ± 0.3	0.9 ± 0.4	1.0 ± 0.3	0
eGFR, mL/min/1.73 m^2^	78.1 ± 23.8	79.5 ± 27.4	76.8 ± 20.5	0
Urea, mg/dL	48.8 ± 20.1	50.1 ± 21.4	47.6 ± 19.1	0
Sodium, mmol/L	139.8 ± 3.1	139.3 ± 3.5	140.3 ± 2.8	0
Potassium, mmol/L	4.3 ± 0.5	4.2 ± 0.5	4.3 ± 0.5	0
Uric acid, mg/dL	6.5 ± 2.5	6.8 ± 3.1	6.2 ± 2.0	0
NT-proBNP, pg/mL	1,615 (640, 2,545)	2,214 (802, 4,180)	1,116 (406, 2,343)	19 (30.7%)
BNP, pg/mL	629 (404, 1,257)	559 (350, 1,298)	629 (419, 1,257)	48 (77.4%)
NPs, standardized^a^	−0.0 ± 1.0	0.3 ± 1.3	−0.3 ± 0.4	5 (8.1%)
UACR, mg/g	31 (10, 73)	30 (6, 172)	32 (15, 66)	16 (25.8%)
VAS, points (0-10)	4.3 ± 2.3	3.8 ± 2.3	4.7 ± 2.2	0
KCCQ-OSS, points (0-100)	64.7 ± 23.2	64.4 ± 23.2	65.0 ± 23.5	0
KCCQ-CSS, points (0-100)	67.3 ± 25.6	67.8 ± 24.1	66.9 ± 27.3	0
ACEi/ARB	24 (39%)	12 (41%)	12 (36%)	0
ACEi/ARB ≥50% rec. dose	13 (54%)	7 (58%)	6 (50%)	
Beta-blocker	38 (61%)	15 (52%)	23 (70%)	0
Beta-blocker ≥50% rec. dose	14 (37%)	6 (40%)	8 (35%)	
Loop diuretic	29 (47%)	13 (45%)	16 (48%)	0
MRA	17 (27%)	7 (24%)	10 (30%)	0

ACEi/ARB = angiotensin-converting enzyme inhibitor/angiotensin receptor blocker; AFib = atrial fibrillation; ARNi = angiotensin-receptor neprilysin inhibitor; BMI = body mass index; CSS = Clinical Summary Score; DBP = diastolic blood pressure; ECG = electrocardiogram; eGFR = estimated glomerular filtration rate; HF = heart failure; KCCQ = Kansas City Cardiomyopathy Questionnaire; LVEF = left ventricular ejection fraction; MRA = mineralocorticoid receptor antagonist; NPs = natriuretic peptides; OSS = Overall Summary Score; SBP = systolic blood pressure; SGLT2i = sodium glucose co-transporter 2 inhibitor; UACR = urinary albumin-to-creatinine ratio; VAS = visual analogic scale.

The number of randomized patients per center was *ULS Gaia/Espinho* n = 19; *ULS Matosinhos* n = 4; *ULS Santo António* n = 16; *ULS São João* n = 23.

a *P* value <0.05.

The trial primary results are presented in the focused *Brief Report* published in the *JACC*.

A description of the study drug doses throughout the study, the use of GDMT at the end of the study, and continuous variation of exploratory parameters is presented in the (Supplemental Tables 1 and 2, and Supplemental Figures 1A to 1E).

A summary of the INITIATE-HFrEF trial design is displayed in the Central Illustration.

**Central Illustration:**
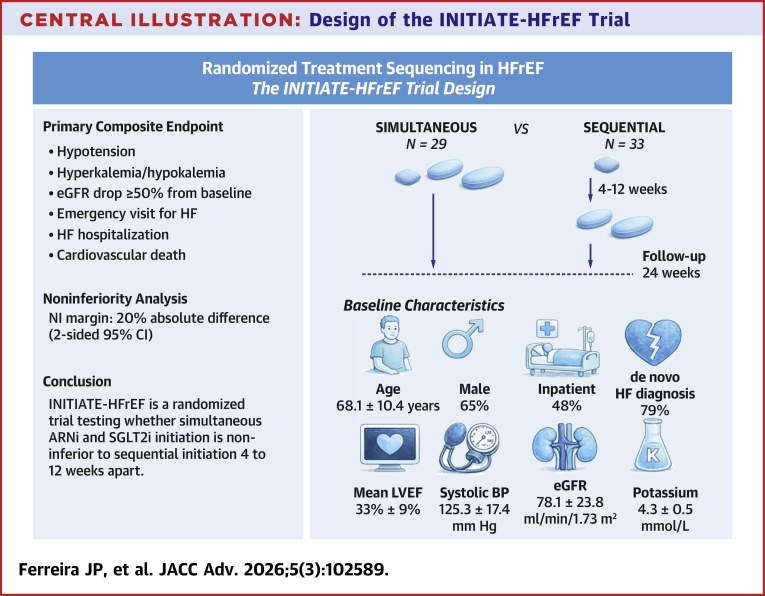
Design of the INITIATE-HFrEF Trial BP = blood pressure; eGFR = estimated glomerular filtration rate; HF = heart failure; LVEF = left ventricular ejection fraction.

## Discussion

The INITIATE-HFrEF randomized trial comparing a simultaneous vs sequential initiation of ARNi and SGLT2i will help informing clinical decisions about the timing of initiation of ARNi and SGLT2i. Importantly, this trial will be relevant to better ascertain whether these therapies can be started simultaneously or if it might be safer to start them sequentially.

The modes of action of ARNi and SGLT2i are distinct and complementary, and both therapies showed early benefit in HFrEF.^9^^,^^15^^,^^16^ Moreover, prior subgroup analysis of HFrEF trials suggested that randomization to SGLT2i on top of sacubitril/valsartan did not modify the benefit of SGLT2i on cardiovascular outcomes and was not associated with apparent excess of adverse events. For example, symptomatic hypotension, hyperkalemia, and acute kidney injury were all reported in <15% of patients using sacubitril/valsartan irrespective of allocation to placebo or SGLT2i.^12^^,^^13^^,^^17^ These subgroup findings are limited due to the nonrandomized chronic use of sacubitril/valsartan; still, they support an independent effect of these agents. “Real-world” data suggest that the combination of both an ARNi and an SGLT2i may be associated with greater clinical benefits than either agent in isolation in propensity-matched diabetic patients with HFrEF.^18^ Therefore, both ARNi and SGLT2i improve the prognosis of patients with HFrEF and their effects seem to occur independently. The INITIATE-HFrEF study placed in the context of these data will further inform if a simultaneous implementation of both therapies is feasible and safe.

Beyond their established benefits for outpatients with HFrEF, both sacubitril/valsartan and SGLT2i showed beneficial effects when added to standard therapy of patients hospitalized for acute HF.^9^^,^^10^^,^^19^ In Portugal (at least in the tertiary centers that participated in the INITIATE-HFrEF trial), the more recent implementation of SGLT2i in patients with “de novo” HF (either as outpatients or inpatients) has been faster than the implementation of ARNi, probably because both dapagliflozin and empagliflozin (the SGLT2i used in INITIATE-HFrEF) are well-tolerated, administered once daily in a single dose, and without the need for up-titration.^20^ To accommodate shifts in clinical practice, we revised the protocol to allow the use of SGLT2i first in the sequential group (as detailed in the Methods section). A rapid initiation and titration of GDMT in HF has the potential to obviate delays in treatment optimization. However, in the recent observational TITRATE-HF (Guideline Implementation and Quality of Care in Patients With Heart Failure) study (NCT06386042), initiation of SGLT2i in newly diagnosed HF was performed in <16% of patients and was frequently the last drug class to be initiated.^21^ Therefore, INITIATE-HFrEF results may better inform strategies of GDMT implementation.

Some limitations should be acknowledged in this study. First, the sample size was substantially smaller than initially planned and, consequently, the study power and precision are decreased. Second, our study is underpowered for assessing “hard” outcomes such as rehospitalization or mortality; however, we know that both SGLT2i and ARNi improve HF outcomes, so the question is more whether it is safe to start them simultaneously. Third, regardless of the trial results, these represent average estimates and do not replace clinical judgment in the context of each individual patient.

## Conclusions

INITIATE-HFrEF is a randomized trial to test whether a simultaneous initiation of ARNi and SGLT2i is noninferior to a sequential initiation of one agent followed by the other 4 to 12 weeks apart.

## Funding support and author disclosures

This study was funded by 10.13039/100004336Novartis. This study was financed by national funds through FCT Fundação para a Ciência e Tecnologia, I.P., under the scope of the Cardiovascular R&D Center – UnIC (UIDB/00051/2020 and UIDP/00051/2020). Drs Oliveira and Pereira-Sousa are supported by FCT PhD Grants (UI/BD/150647/2020, UI/BD/154953/2023, and UI/BD/154952/2023). Dr Saraiva is funded by national funds through FCT – Fundação para a Ciência e a Tecnologia, I.P., and by the Recovery and Resilience Plan (PRR), in the scope of the “Ciência Mais Capacitação,” under the FCT-Tenure programme (2023.13694.TENURE.021). The authors have reported that they have no relationships relevant to the contents of this paper to disclose.
