# Supplementary material for: Design, Rationale, and Methods of the INITIATE-HFrEF Trial: ARNI and SGLT2 Inhibitors Sequencing in HFrEF
Source: JACC Adv. 2026 Feb 4;5(3):102589. doi: 10.1016/j.jacadv.2026.102589 (PMC13100719; doi:10.1016/j.jacadv.2026.102589)
Supplement: Supplemental material [file mmc1.pdf]

Supplementary Material

[$] Adriana Costa, MD[6]; Ana Beatriz Batista, MSc[1,4]; Ana Filipa Ferreira, PhD[1]; Ana Margarida Fonseca, MD[6]; Ana Sofia Correia, MD[8]; Cândida Gonçalves, MSc[7]; Catarina Faustino, MD[6]; Catarina Gomes, MD[7]; Catarina Pereira, MD[3]; Diogo Ferreira, MD[1,5]; Fernando Friões, MD, PhD[1,6]; Giulia Rinaldi, BSc[1]; Gualter Silva, MD[5]; Joana Mascarenhas, MD[3]; Jorge Almeida, MD[6]; Maria Isilda Oliveira, MSc[8]; Mariana Taveira, MD[8]; Pedro Marques, MD[1,6]; Pedro Palma, MD[12]; Sara Henriques, MD[3]; Sofia Moreira-Silva, MD[8]; Vânia Rocha, MSc[8]

Supplementary Table 1. Study treatments and doses at study visits

SGLT2i	Week 4 (N =56 on SGLT2i)			
	-	No	Dapa.	Empa.
Baseline (N =53 on SGLT2I)	No	2	3	0
	Dapa.	0	37	0
	Empa.	0	0	16
	Week 12 (N =58 on SGLT2i)			
	-	No	Dapa.	Empa.
	No	0	3	2
	Dapa.	0	37	0
	Empa.	0	0	16
	Week 24 (N =58 on SGLT2i)			
	-	No	Dapa.	Empa.
	No	0	3	2
	Dapa.	0	37	1
	Empa.	0	1	15
ARNi	Week 4 (N =55 on ARNi)			
	-	No	Yes	-
Baseline (N =35 on ARNi)	No	3	24	-
	Yes	0	31	-
	Week 12 (N =55 on ARNi)			
	-	No	Yes	-
	No	2	23	-

	Yes	1	32	-
	Week 24 (N =55 on ARNi)			
	-	No	Yes	-
	No	2	24	-
	Yes	1	32	-

Legend: SGLT2i, sodium-glucose co-transporter 2 inhibitor; ARNi, angiotensin-receptor neprilysin inhibitor.

ARNi dose at week 4: 24/26 mg bid N=35 (63.6%); 49/51 mg bid N =19 (34.5%); 97/103 mg bid N =1 (1.8%).

ARNi dose at week 12: 24/26 mg bid N=25 (45.5%); 49/51 mg bid N =19 (34.5%); 97/103 mg bid N =11 (20%).

ARNi dose at week 24: 24/26 mg bid N=20 (36.4%); 49/51 mg bid N =23 (41.8%); 97/103 mg bid N =12 (21.8%).

Note: Throughout the study 5 patients in the sequential group did not receive ARNi due to death (n =1), physician decision (n =2), study withdrawal (n =2); 2 patients in the simultaneous group did not receive ARNi due to death (n =1) and physician decision (n =1). Patients who died or withdrew from the study also did not receive SLGT2i (n =4).

Supplementary Table 2. Use of GDMT at the end of the study

GDMT	Simultaneous<br>n/N (%)	Sequential<br>n/N (%)
Beta-blocker	26/28 (92.9)	28/30 (93.3)
Beta-blocker ≥50% rec. dose	19/26 (73.1)	24/28 (85.7)
MRA	19/28 (67.9)	23/30 (76.7)

Legend: GDMT, guideline-directed medical therapy; MRA, mineralocorticoid receptor antagonist.

Supplementary Figure 1. Treatment allocation and influence on other markers

A) Weight

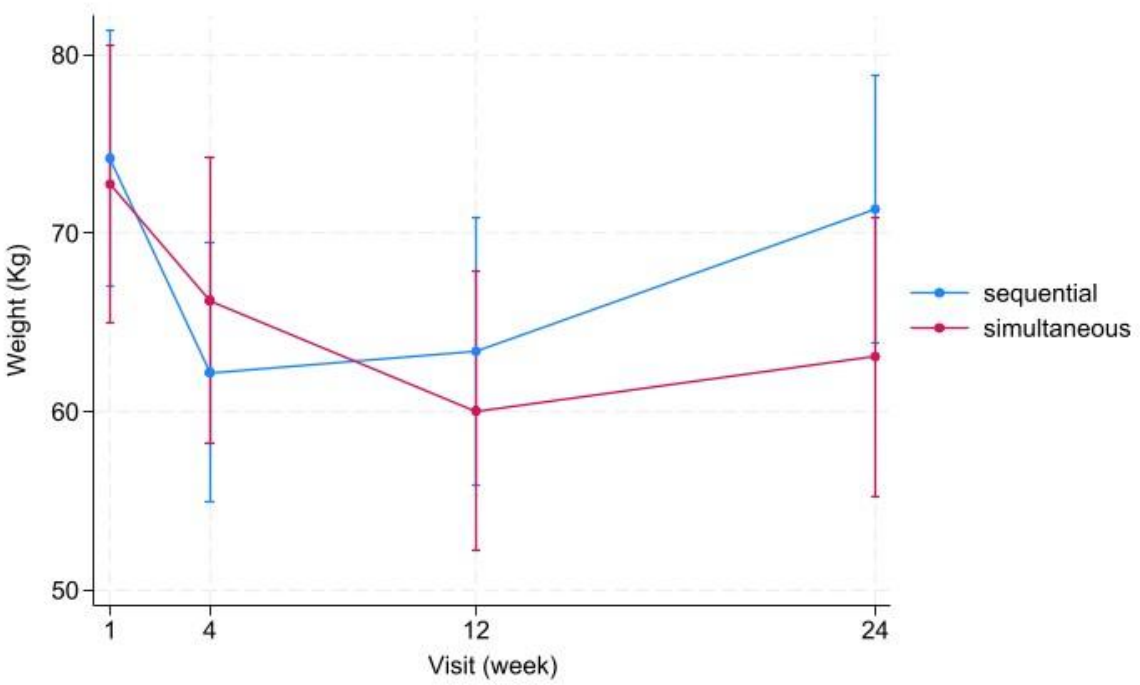

B) Uric acid

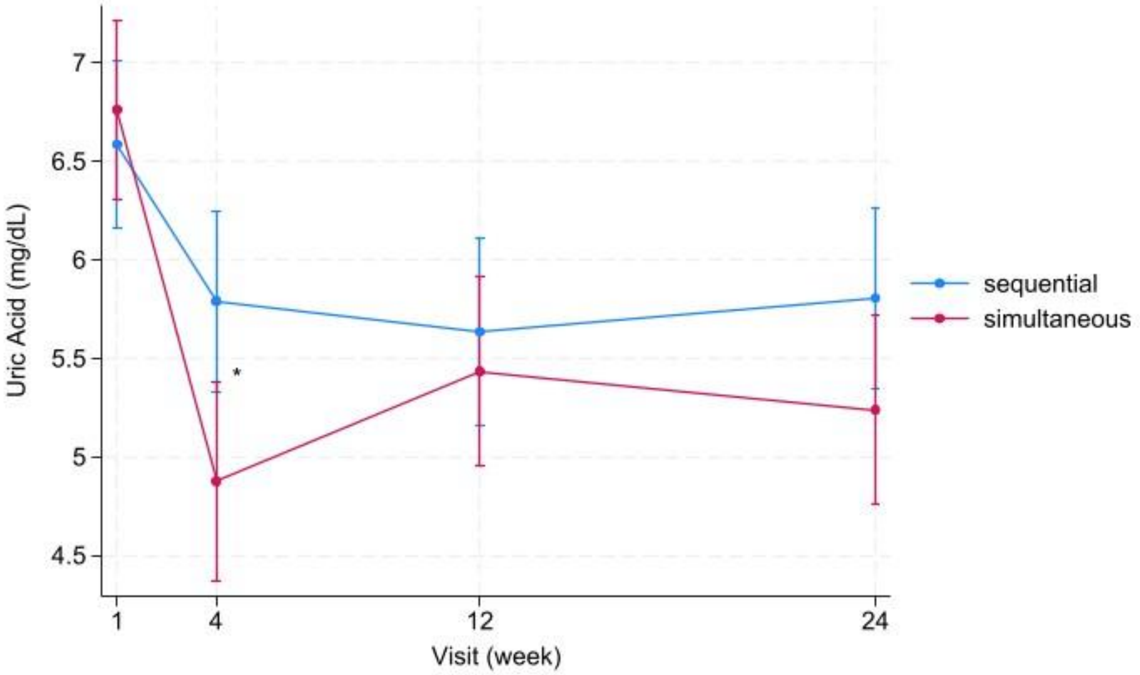

C) Hemoglobin

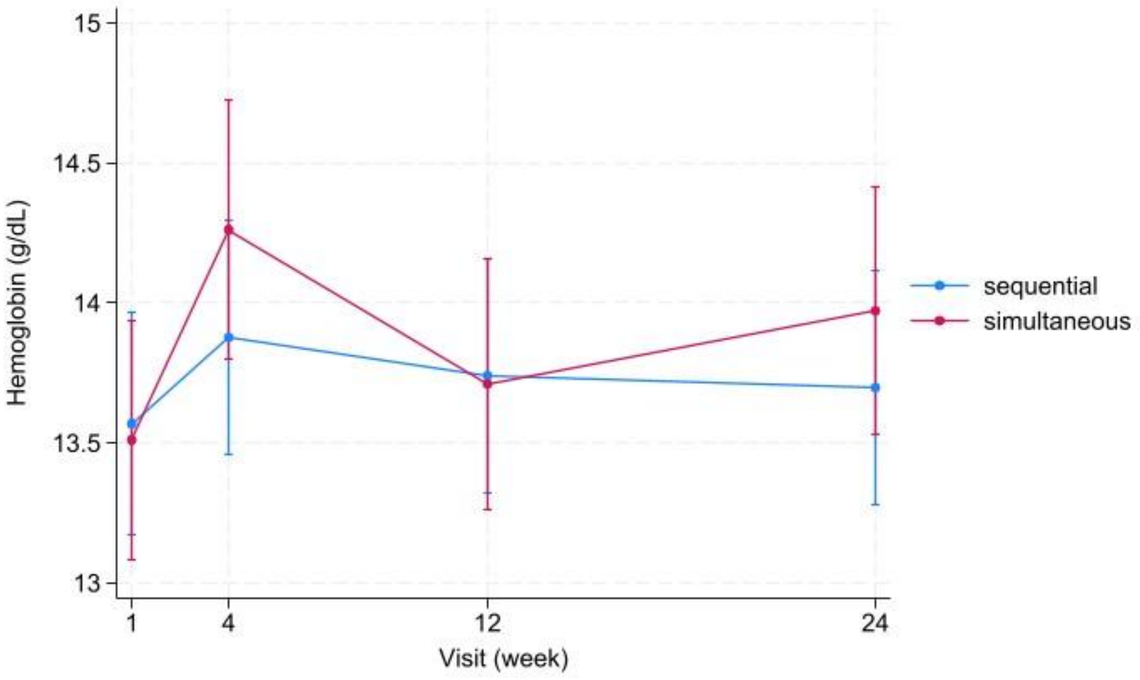

D) KCCQ-CSS

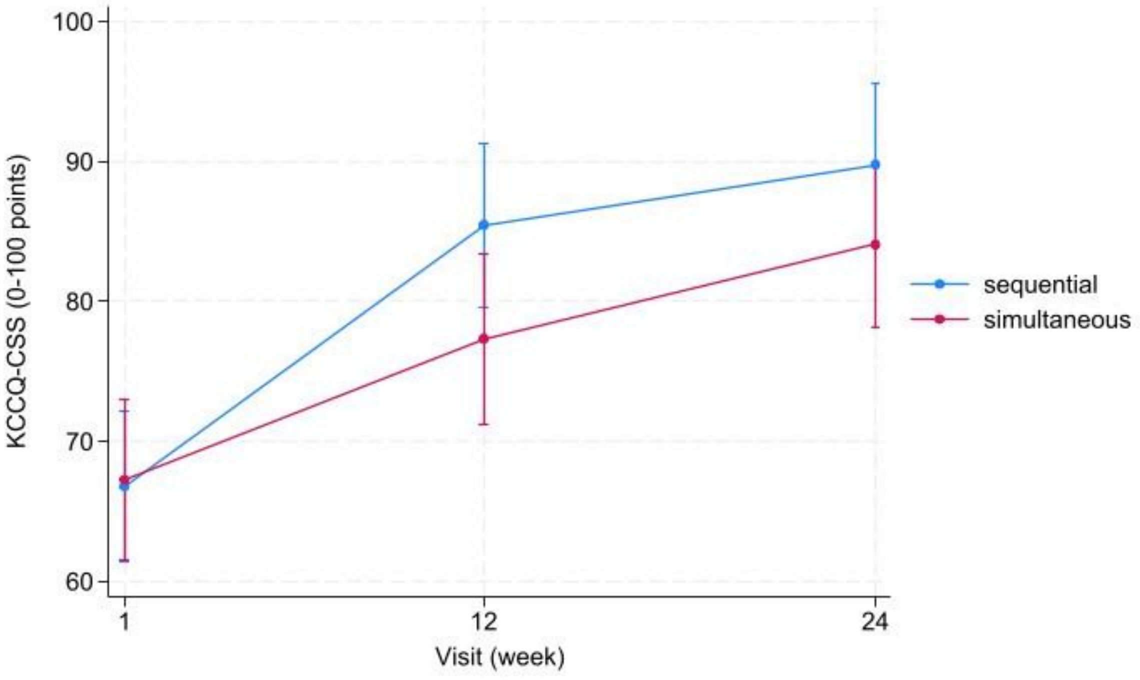

E) Natriuretic peptides

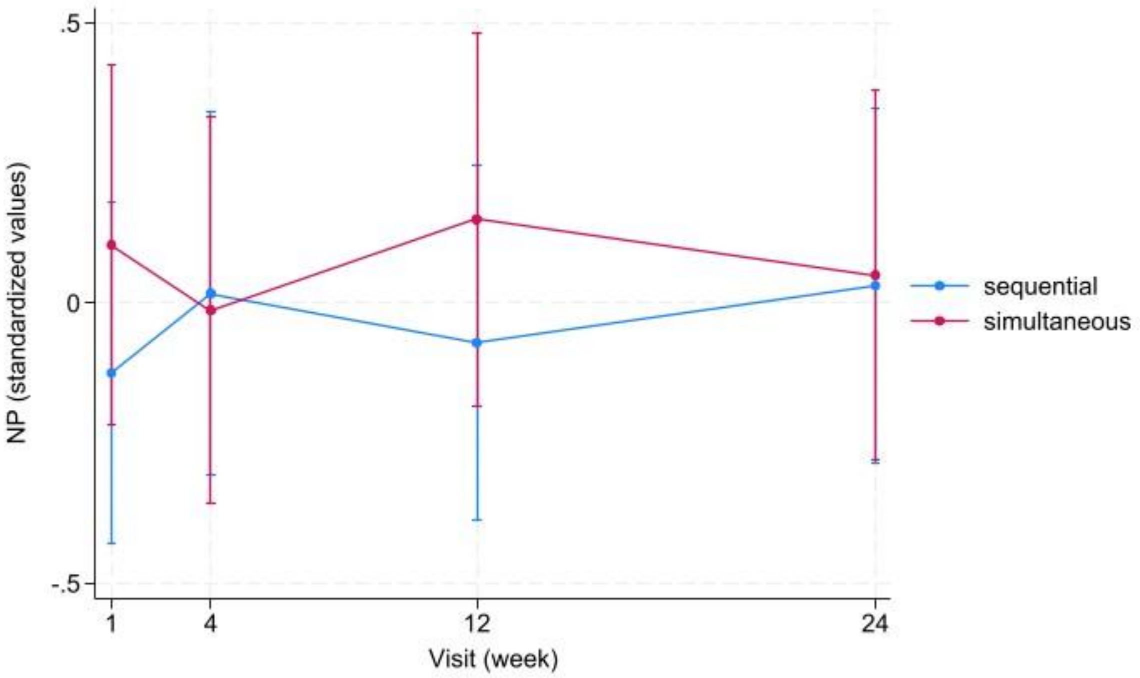

Legend: VAS, visual analogic scale; KCCQ-CSS, Kansas City Cardiomyopathy Questionnaire-Clinical Summary Score.

Change (95%CI) from baseline in: A) Weight in Kg, week 4 =-9.4 (-15.2 to -3.6), week 12 =-11.7 (-17.5 to -5.9), week 24 =-6.1 (-11.8 to -0.3); B) Uric acid in mg/dL, week 4 =-1.3 (-1.7 to -0.8) *more pronounced reduction in the simultaneous =-1.8 (-2.4 to -1.2) vs sequential group =-0.7 (-1.3 to -0.2), week 12 =-1.1 (-1.5 to -0.7), week 24 =1.1 (-1.5 to -0.7); C) Hemoglobin in g/dL, week 4 =0.5 (0.2 to 0.9), week 12 =0.2 (-0.1 to 0.6), week 24 =0.3 (-0.1 to 0.7); D) KCCQ-CSS in points, week 12 =15 (9 to 20), week 24 =20 (14 to 26); E) Natriuretic peptides in standardized values, week 4 =0.01 (-0.22 to 0.25), week 12 =0.04 (-0.19 t0 0.28), week 24 =0.05 (-0.18 to 0.28).
